# Distribution and Characteristics of Human Plague Cases and *Yersinia pestis* Isolates from 4 *Marmota* Plague Foci, China, 1950–2019

**DOI:** 10.3201/eid2710.202239

**Published:** 2021-10

**Authors:** Zhaokai He, Baiqing Wei, Yujiang Zhang, Jun Liu, Jinxiao Xi, Dunzhu Ciren, Teng Qi, Junrong Liang, Ran Duan, Shuai Qin, Dongyue Lv, Yuhuang Chen, Meng Xiao, Rong Fan, Zhizhong Song, Huaiqi Jing, Xin Wang

**Affiliations:** National Institute for Communicable Disease Control and Prevention, Beijing, China (Z. He, J. Liang, R. Duan, S. Qin, D. Lv, M. Xiao, R. Fan, H. Jing, X. Wang);; Qinghai Institute for Endemic Disease Control and Prevention, Xining, China (B. Wei);; Center for Disease Control and Prevention of Xinjiang Uygur Autonomous Region, Urumqi, China (Y. Zhang);; Inner Mongolia Autonomous Region Comprehensive Center for Disease Control and Prevention, Hohhot, China (J. Liu);; Gansu Provincial Centre for Disease Control and Prevention, Lanzhou, China (J. Xi);; Center for Disease Control and Prevention of Tibet Autonomous Region, Lhasa, China (D. Ciren);; Sichuan Center for Disease Control and Prevention, Chengdu, China (T. Qi);; Shenzhen Nanshan Maternity and Child Healthcare Hospital, Shenzhen, China (Y. Chen);; Yunnan Center for Disease Control and Prevention, Kunming, China (Z. Song)

**Keywords:** *Marmota*, plague, marmots, *Yersinia pestis*, epidemiology, bacteria, vector-borne infections, fleas, parasites, public health, zoonoses, China

## Abstract

We analyzed epidemiologic characteristics and distribution of 1,067 human plague cases and 5,958 *Yersinia pestis* isolates collected from humans, host animals, and insect vectors during 1950–2019 in 4 *Marmota* plague foci in China. The case-fatality rate for plague in humans was 68.88%; the overall trend slowly decreased over time but fluctuated greatly. Most human cases (98.31%) and isolates (82.06%) identified from any source were from the *Marmota himalayana* plague focus. The tendency among human cases could be divided into 3 stages: 1950–1969, 1970–2003, and 2004–2019. The *Marmota sibirica* plague focus has not had identified human cases nor isolates since 1926. However, in the other 3 foci, *Y. pestis* continues to circulate among animal hosts; ecologic factors might affect local *Y. pestis* activity. *Marmota* plague foci are active in China, and the epidemic boundary is constantly expanding, posing a potential threat to domestic and global public health.

Plague is a highly virulent fleaborne zoonotic disease caused by the bacterium *Yersinia pestis* (*1*,*2*). Humans can acquire plague through contact with infectious animal tissues or through inhalation. Human contact with infectious animal tissues usually occurs when hunting, trapping, skinning, or handling meat of infected animals. Humans infected through animal contact can develop pneumonic plague and, if not treated, spread their infections to other persons by coughing infectious respiratory droplets. Over the course of human history, plague pandemics have caused hundreds of millions of deaths around the world (*3*–*5*). Currently, the Democratic Republic of the Congo and Madagascar in Africa are the most severe plague epidemic areas; human cases are reported in Madagascar almost every year (*6*–*10*). Plague outbreaks also have caused major public health crises in China. In the first half of the 20th Century, *Y. pestis* caused several large epidemics and nearly 1 million deaths (*11*–*17*).

Plague foci in China are divided into 12 types on the basis of geographic landscape, host, vector, and *Y. pestis* ecotype characteristics (*18*). Among these, 4 are *Marmota* (marmot) foci: the *Marmota himalayana* (Himalayan marmot) plague focus, located in the Qinghai-Tibet Plateau; the *Marmota baibacina*–*Spermophilus undulatus* (gray marmot–long-tailed ground squirrel) plague focus, in the Tianshan Mountains; the *Marmota caudata* (red marmot) plague focus, in the Pamir Plateau; and the *Marmota sibirica* (Tarbagan marmot) plague focus, in the Hulun Buir Plateau of Inner Mongolia. 

The *Marmota himalayana* plague focus is the largest and the most active foci in China. This focus covers >443,290 km^2^ and, before the 1990s, most human cases occurred here. Since the 1990s, rat-associated plague epidemics have erupted in southern China, but beginning in 2004, the *Marmota himalayana* plague focus re-emerged as the main source of human cases. Outbreaks have occurred every few years in this focus. 

Ecologic factors, including relevant environmental variables, have strong impacts on the shift between periods of inactivity, when plague is maintained at low levels of transmission among its animal reservoirs, and periods of activity, when rates of transmission greatly increase and cause widespread die-offs among susceptible rodents and increased numbers of human cases (*19*–*22*). Because of limitations of experimental technology and economic conditions, plague cases before 1958 were confirmed only by clinical symptoms, such as sudden high fever and lymphadenopathy; human cases usually were accompanied by increased rates of rodent deaths in the area before or at the early stage of human illnesses. Since the 1950s, China has strengthened plague surveillance and control and the number of human cases has decreased rapidly. Cases in the *Marmota* plague foci have continued to slowly decline; nevertheless, the combined numbers of cases from these foci during 1950–2019 exceeded the total number of plague cases in the United States during 1900–2012 (*23*).

Currently, most human cases in China occur in the *Marmota* plague foci. We investigated the distribution and characteristics of human plague cases and *Y. pestis* isolates recovered from animal hosts (mainly marmots) and insect vectors (mainly fleas) during 1950–2019. We describe the prevalence of *Y. pestis* in humans, animal hosts, and insect vectors of China. 

## Methods

### Data Sources and Analyses

**Figure 1:**
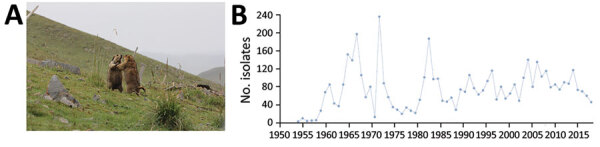
Plague ecology and surveillance of *Yersinia pestis* in the *Marmota himalayana* plague focus, Qinghai-Tibet Plateau, China, 1950–2019. This focus area encompasses Qinghai Province, Gansu Province, Tibet Autonomous Region, Sichuan Province, and Xinjiang Uygur Autonomous Region. A) The Himalayan marmot (*M. himalayana*), the predominant marmot species in this focus. Photograph by Xin Wang. B) Number of *Y. pestis* isolates collected from humans, animal hosts, and insect vectors (mostly *Callopsylla dolabris* and *Oropsylla silantiewi* fleas) in the focus.

**Figure 4:**
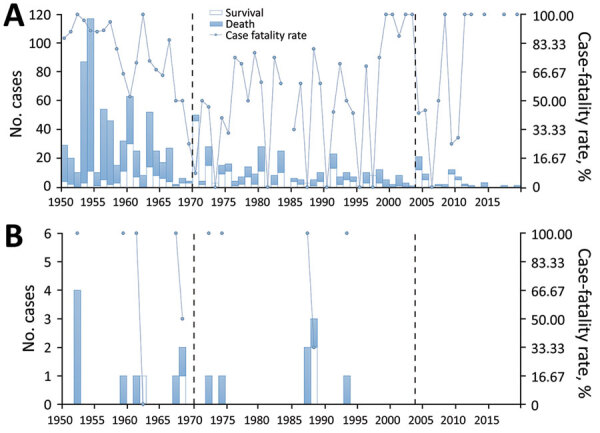
Frequency of human plague cases and case-fatality rates in 2 *Marmota* plague foci, China, 1950–2019. A) *Marmota himalayana* plague focus of the Qinghai-Tibet Plateau, which includes Qinghai Province, Gansu Province, Tibet Autonomous Region, Sichuan Province, and Xinjiang Uygur Autonomous Region. B) *Marmota baibacina–Spermophilus undulatus* plague focus includes Xinjiang Uygur Autonomous Region.

**Figure 5:**
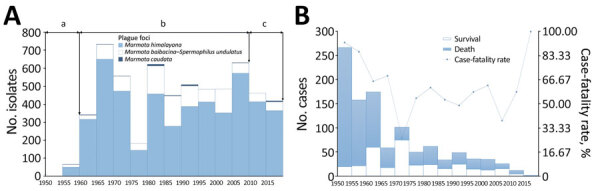
Number of *Yersinia pestis* isolates and human plague cases in *Marmota* plague foci, China, 1950–2019. Columns represent 5-year intervals. The 3 plague foci from which *Y. pestis* isolates have been collected are the *Marmota himalayana* plague focus of the Qinghai-Tibet Plateau, which includes Qinghai Province, Gansu Province, Tibet Autonomous Region, Sichuan Province, and Xinjiang Uygur Autonomous Region; the *Marmota baibacina–Spermophilus undulatus* plague focus of the Tianshan Mountains, Xinjiang Uygur Autonomous Region; and the *Marmota caudata* plague focus of the Pamir Plateau, Xinjiang Uygur Autonomous Region. A) Number of *Y. pestis* isolates collected from humans, animal hosts, and insect vectors. Lowercase letters at top indicate periods of isolate collection: a) early attempts during 1950–1959; b) increased diagnosis and animal plague surveillance increased number isolates collected during 1960–2009; and c) decrease in isolates likely due to decreasing numbers of dead marmot species found around active *Y. pestis* areas during 2010–2019. B) Number of human plague cases and case-fatality rates.

We included human cases and *Y. pestis* isolates from *Marmota* plague foci in China collected during 1950–2019. We applied descriptive statistics to analyze the distribution of isolates and human cases for each year (Figure 1–4) and for 5-year intervals (Figure 5). The main sources of data were 2 texts on the history of plague in China (*24*,*25*) and surveillance data of plague obtained from the regions comprising the *Marmota* plague foci, namely Qinghai Province (Qinghai), Gansu Province (Gansu), Tibet Autonomous Region (Tibet), Sichuan Province (Sichuan), Xinjiang Uygur Autonomous Region (Xinjiang), and Inner Mongolia Autonomous Region (Inner Mongolia) (*26*). These foci contain vast lands away from human habitation and surveillance efforts over time have gradually focused more intensely on the spots within these foci where plague is prevalent in marmots and found near human habitations.

The sources of *Y. pestis* isolates in *Marmota* plague foci were animals found dead in the environment, plague patients, insect vectors, and a few live animals. Samples collected from animals found dead in the environment showed most animals died of plague.

### Diagnosis of Human Plague Cases

Human cases before 1958 included in the study mainly were confirmed by clinical and epidemiologic investigation based on symptoms, as stated. Human cases after 1958 included in the study had been confirmed by microbiological or serologic diagnosis.

## Results

### Landscape, Host, Vector, and Other Ecologic Features

**Figure 6:**
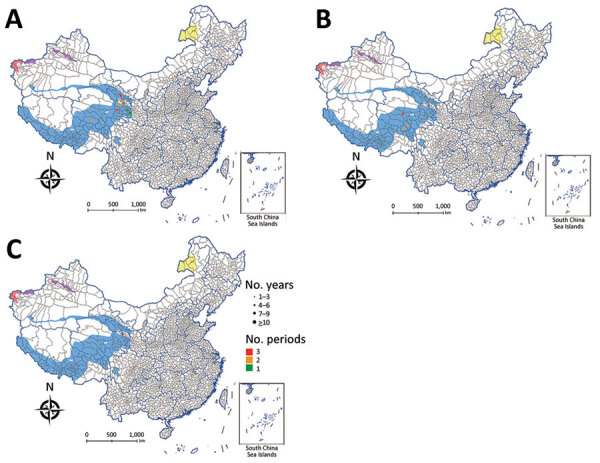
Human plague cases detected in 4 *Marmota* plague foci, China. A) 1950–1969; B) 1970–2003; C) 2004–2019. Dot size indicates the number of years during which plague occurred in each timeframe; dot colors indicates number of periods (A, B, C) during which plague occurred for each location. Blue shading indicates the *Marmota himalayana* plague focus; purple shading indicates the *Marmota baibacina–Spermophilus undulatus* plague focus; red shading indicates the *Marmota caudata* plague focus; and yellow shading indicates the *Marmota sibirica* plague focus.

The *Marmota himalayana* plague focus, identified in 1954, covers Qinghai, Gansu, Tibet, Sichuan, and Xinjiang (Figure 6). The main host is the Himalayan marmot (Figure 1, panel A), which inhabits high-frigid shrub habitats and meadow-steppe zone at altitudes of 2,700–5,450 m. *Callopsylla dolabris* and *Oropsylla silantiewi* fleas are the main insect vectors (Table 1).

**Table 1:** Ecologic characteristics of *Marmota* plague foci, China, 1950–2019

Characteristics	Marmota himalayana*	*Marmota baibacina–Spermophilus undulatus*†	*Marmota caudata*‡	*Marmota sibirica*§
Year *Yersinia pestis* first isolated	1954	1955	1956	1923
Regions	Qinghai Province, Gansu Province, Tibet Autonomous Region, Sichuan Province, Xinjiang Uygur Autonomous Region	Xinjiang Uygur Autonomous Region	Xinjiang Uygur Autonomous Region	Inner Mongolia Autonomous Region
Hosts	Himalayan marmot	Gray marmot, long-tailed ground squirrel	Red marmot	Tarbagan marmot
Vectors	*Callopsylla dolabris*,* Oropsylla silantiewi *fleas	*O. silantiewi, Citellophilus tesquorum *fleas	*O. silantiewi *fleas	*O. silantiewi *fleas
Altitude	2,700–5,450 m	1,600–4,000 m	2,800–5,000 m	600–800 m
Hibernation				
Start	September–October	September	Mid-September	Late September–early October
End	Late March–mid-April	Early- to mid-April	Early April	Late March–early April
Animal plague season	April–October	May-September	May–August	NA¶
Peak	June–July	July	July	NA¶
Habitat	High-frigid shrubs, meadow-steppe	Forest-meadow-steppe, alpine meadow-steppe	Alpine steppe	Low mountains and hills, meadow-steppe

*Qinghai-Tibet Plateau. †Tianshan Mountains. ‡Pamirs Plateau. §Hulun Buir Plateau of Inner Mongolia. ¶No *Y. pestis* isolated since 1926.

**Figure 2:**
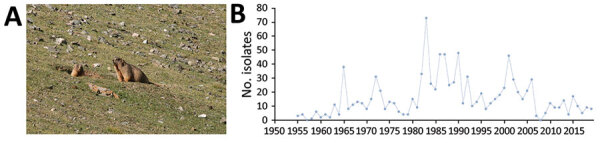
Plague ecology and surveillance of *Yersinia pestis* in the *Marmota baibacina–Spermophilus undulatus* plague focus of the Tianshan Mountains, Xinjiang Uygur Autonomous Region, China, 1950–2019. A) The gray marmot (*M. baibacina*), the predominant marmot species in this focus. Photograph by Yujiang Zhang. The long-tailed ground squirrel (*S. undulatus*) also is an *Y. pestis* host in the focus. B) Number of *Y. pestis* isolates collected from humans, animal hosts, and insect vectors (mostly *Oropsylla silantiewi* and *Citellophilus tesquorum* fleas) in the focus.

The *Marmota baibacina–Spermophilus undulatus* plague focus was identified in 1955 and is in Xinjiang (Figure 6). The main animal hosts are the gray marmot (Figure 2, panel A) and long-tailed ground squirrel, both of which inhabit forest meadow-steppe and alpine meadow-steppe zones at altitudes of 1,600–4,000 m. *O. silantiewi* fleas and *Citellophilus tesquorum* fleas are the main vectors.

**Figure 3:**
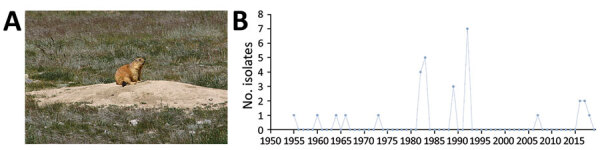
Plague ecology and surveillance of *Yersinia pestis* in the *Marmota caudata* plague focus of the Pamir Plateau, Xinjiang Uygur Autonomous Region, China, 1950–2019. A) The red marmot (*M. caudata*), the predominant marmot species in this focus. Photograph by Yujiang Zhang. B) Number of *Y. pestis* isolates collected from humans, animal hosts, and insect vectors (mostly *Oropsylla silantiewi* fleas) in the focus.

The *Marmota caudata* plague focus was identified in 1956 and is located in Xinjiang (Figure 6). The main host is the red marmot, also known as the golden or long-tailed marmot (Figure 3, panel A), which inhabits the alpine steppe zone at altitudes of 2,800–5,000 m, but most live in the higher end of the range at 3,800–5,000 m. *O. silantiewi* fleas are the main insect vector.

**Figure 7:**
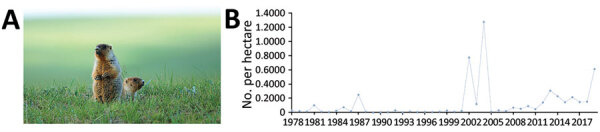
Ecology and surveillance of marmots in the *Marmota sibirica* plague focus of the Hulun Buir Plateau, Inner Mongolia, 1950–2019. A) Tarbagan marmot (*M. sibirica*), the predominant marmot species in this focus. Photograph by Jun Liu. B) Average density of this species in the focus area. Tarbagan marmots host *Oropsylla silantiewi* fleas, a known vector of *Yersinia pestis*, but no *Y. pestis* isolate has been collected from humans, animal hosts, or insect vectors in this focus since 1926.

The *Marmota sibirica* plague focus is located at the border of China, Mongolia, and Russia. The focus, located in Inner Mongolia (Figure 6), was identified in 1923. The main animal host is the Tarbagan marmot (Figure 7, panel A), which inhabits low mountains and hills and the meadow-steppe zone at altitudes of 600–800 m. *O. silantiewi* fleas are the main insect vector in this focus. No *Y. pestis* strains have been isolated from the *Marmota sibirica* plague focus since 1926; we only measured host surveillance in this focus. Surveillance of the Tarbagan marmot population density was started in 1978 (Figure 7, panel B). Professionals from the local Chinese Center for Disease Control and Prevention office conduct surveillance by counting the number of marmots observed along survey routes, then calculate density by dividing the number of marmots observed by the size of area. During 1978–2019, the density of *M. sibirica* in the focus area was highest in 2004, 1.274 marmots/hectare (10,000 m^2^) and lowest in 1989 (0 per hectare) (Figure 7, panel B).

### Distribution Characteristics of *Y. pestis* Isolates

During 1950–2019, a total of 5,958 *Y. pestis* isolates were recovered from *Marmota* plague foci in China. On average, 85.11 *Y. pestis* isolates were recovered each year. The years with the most isolates were 1967 (208 isolates), 1983 (265 isolates), and 1972 (267 isolates). No *Y. pestis* isolates were recovered from the *Marmota sibirica* plague focus during this period.

*Y. pestis* has been isolated in the *Marmota himalayana* plague focus every year since the first isolate was recovered in 1954. During 1954–2019, 4,889 isolates were collected from the focus. In the *Marmota baibacina–Spermophilus undulatus* plague focus, *Y. pestis* isolates were first recovered in 1955 and a total of 1,039 isolates were collected by 2019. In the *Marmota caudata* plague focus, *Y. pestis* was first isolated in 1956 and 30 isolates were collected by 2019.

### Epidemiologic Characteristics of Human Plague Cases

During 1950–2019, total of 1,067 human plague cases have occurred in the 4 *Marmota* plague foci in China, including 735 deaths. The average case-fatality rate was 68.88%, 15.24 cases annually. Among all plague cases, 690 were confirmed by laboratory or serologic diagnosis; 377 were diagnosed by clinical and epidemiologic investigations. Most cases occurred in 1953 (87 cases), 1954 (117 cases), and 1960 (63 cases). 

During 1950–2019, the *Marmota himalayana* plague focus had the most (1,049) human cases (Figure 4, panel A); only 18 cases have occurred in the *Marmota baibacina–Spermophilus undulatus* plague focus (Figure 4, panel B). No human cases have been reported in the *Marmota caudata* or *Marmota sibirica* plague foci during 1950–2019.

Human case counts during 5-year intervals show the highest number of cases (267) occurred during 1950–1954 and the lowest (2 cases) during 2015–2019. Mortality rates were highest (92.51%) during 1950–1954 and 2015–2019 (100%) and lowest (26.47%) during 1970–1974 (Figure 5, panel B). 

The patterns of decline for human cases are different in the 2 most active foci (Figure 4). In the *Marmota himalayana* plague focus, the number of cases was relatively high and periodically fluctuated, but both the number and frequency of peaks declined over time (Figure 4, panel A). During 1950–1969, the number of cases was high and the peak interval was short; during 1970–2003, the number of cases decreased sharply and the interval was longer; after 2004, the number of cases declined further and in some years no cases were reported. In contrast, the pattern of decline in human cases in the *Marmota baibacina–Spermophilus undulatus* plague focus has had longer intervals. In several years <4 human cases occurred and no case has been reported since 1994 (Figure 4, panel B). During 1950–2019, primary and secondary pneumonic plague accounted for 69.26% (739/1,067) of cases, among which the case-fatality rate was 75.10% (555/739) (Table 2). The rate of other plague types, such as bubonic and septicemic, was 54.88% (180/328) (Table 2).

**Table 2:** Case-fatality rates for pneumonic and other types of plague, by *Marmota* plague focus, China, 1950–2019*

Focus	Pneumonic plague	Other plague types†	Total
*Marmota himalayana*‡	75.03 (544/725)	54.63 (177/324)	68.73 (721/1,049)
*Marmota baibacina–Spermophilus undulatus*§	78.57 (11/14)	75.00 (3/4)	77.78 (14/18)
*Marmota caudata*¶	0	0	0
*Marmota sibirica*#	0	0	0
Total case-fatality rate	75.10 (555/739)	54.88 (180/328)	68.88 (735/1,067)

*Data are reported as case-fatality rate% (no. deaths/no. cases). †Including bubonic, septicemic, and other types. ‡Qinghai-Tibet Plateau. §Tianshan Mountains. ¶Pamirs Plateau. #Hulun Buir Plateau of Inner Mongolia.

## Discussion

Four *Marmota* plague foci have been identified and classified in China. Each focus is characterized by the marmot species that serves as the primary *Y. pestis* host in the region, but other animals in these areas also can be infected, usually with highly virulent *Y. pestis* strains (*27*). The 4 foci are active areas where animal and human plague epidemics continue in China. Except for the *Marmota sibirica* plague focus, most foci are in western China (Figure 6). Climatic conditions, such as long winters with heavy snow, are characteristic of these foci, as are unique geographic features, such as frozen glacial soils, cold desert plateaus, alpine grasslands, and river wetlands. Many rare wild animals inhabit these plateaus, and the human population is sparse. Little human disturbance and relatively stable plague ecology could be conducive to maintaining the ecologic balance and active plague status of these foci (*21*). 

The *Marmota sibirica* plague focus is located at the junction of China, Mongolia, and Russia, and 2 plague epidemics occurred in this focus in the early 20th Century. Of note, no human case has been reported in this part of China since 1923, and *Y. pestis* has not been isolated from there since 1926. However, cases of animal and human plague from this focus are reported in Mongolia and Russia (*11*–*17*,*28*–*35*). The main reason for the lack of human cases in this focus in China is the considerable decrease in Tarbagan marmot populations, which are believed to be due to ecologic changes and increased human activity. The development of animal husbandry, such as raising sheep that graze in marmot habitats in the region, and environmental disruption due to transportation and farming activities, have caused irreversible damage to the Tarbagan marmot habitat. In addition, sale of marmot skins and meat have depleted the population; ≈2,500,000 marmot skins were purchased in 1910, the peak year. In addition, a large fire in 1987 caused devastating damage to the Tarbagan marmot habitat and killed ≈200,000 marmots (*36*). 

When the existing ecologic balance is disrupted, for instance due to habitat damage, the number of *Y. pestis* hosts and the host density changes, thus affecting the survival of the bacterium*. Y. pestis* circulates among animal and vector hosts within a limited range and with discrete distribution where it seldom causes human plague and is rarely detected by regular surveillance. The interactions between host, pathogen, and environment seem to be gradually settling into a period of relative inactivity in all *Marmota* plague foci (*19*,*21*).

China began attempts to isolate *Y. pestis* during 1950–1959 and the number of isolates recovered was relatively low (Figure 5). During 1960–2009, the number of collected isolates greatly increased, mainly due to improved case diagnosis and animal plague surveillance and plague diagnosis is confirmed through pathogen isolation or other laboratory evidence. During 2010–2019, the number of *Y. pestis* isolates decreased in China, which seems to be related to the decrease of marmots found dead around active plague locations and human cases. In 1953, China established specialized plague investigation agencies for the *Marmota* plague foci. Many unsuccessful attempts at eradicating *Y. pestis* in these foci have demonstrated that plague is inherent in these areas and cannot be eliminated by large scale attempts to kill rodent hosts (*21*,*27*,*37*). 

After these unsuccessful attempts to reduce the possibility of human exposure to plague, *Y. pestis* elimination efforts have focused more on key locations and key seasons, particularly in locations with epidemic activity and during the peak marmot and flea breeding seasons (*38*). Long-term training and education among clinicians has resulted in more timely diagnosis, treatment, and reporting of human plague cases (*39*). Improving humans’ awareness about plague and their ability to protect themselves seems to have reduced their exposure to marmots and infected fleas. For future surveillance, appropriate typing methods for tracking the spread of certain *Y. pestis* strains are needed. The *Y. pestis* genome remains stable in *Marmota* plague foci, enabling *Y. pestis* to maintain its high pathogenicity and virulence, and most *Y. pestis* strains in the *Marmota* plague foci are highly virulent (*27*). Single-nucleotide polymorphism typing and other phylogenic studies have shown that *Y. pestis* clusters geographically in China; it could take years to accumulate several mutations within the same focus or region (*40*).

During 1950–2019, human cases in the *Marmota* plague foci of China were mainly concentrated in the *Marmota himalayana* focus, which has remained active since it was identified in 1954 (*41*,*42*). Only 18 human cases have been reported in the *Marmota baibacina–Spermophilus undulatus* plague focus. No human case has ever been reported in the *Marmota caudata* plague focus since it was identified in 1956, which can be attributed to limited human habitation and limited exposure to infected animals. No human cases have been reported in the *Marmota sibirica* plague focus since 1923; marmot density in this region generally is low but has been reported as being abnormally high for several years (Figure 7, panel B), perhaps because earlier sampling was not representative of the whole because survey areas were limited. Recent ecologic protection measures in this region might raise the Tarbagan marmot density, which could increase the numbers of *Y. pestis*–infected marmots. Previously scattered pockets of *Y. pestis* infection might enlarge and eventually merge with other pockets, leading to a threat of increased spread among marmots and heightened human plague risk (*19*,*21*).

Human cases in the *Marmota* plague foci have shown a decreasing trend, but a slower decrease than in other plague foci of China. *Marmota* plague foci had a relatively high average case-fatality rate of 68.88% during 1950–2019 because of a higher proportion of pneumonic cases, which had a much higher average case-fatality rate (75.10%) than other plague types. In contrast, bubonic and septicemic plague had a combined case-fatality rate of 54.88%. Because pneumonic plague can be transmitted directly between humans without vector involvement (*37*,*43*), it can spread rapidly in densely populated areas, as has occurred in several pandemics (*3*–*5*,*44*). In China, human cases before 1958 were pneumonic plague and had an extremely high fatality rate of 92.57% (Figure 5, panel B). Delayed medical treatment and misdiagnosis might contribute to high fatality rates. For instance, when horses were the main form of transportation, most human cases progressed to pneumonic plague because timely access to treatment was not possible; large-scale outbreaks with human-to-human transmission and high case-fatality rates were reported. At the same time, some sporadic or bubonic cases might have been missed. Nevertheless, the possibility of misdiagnosis in the past is estimated to be low because of high case-fatality rates, obvious clinical features, and epidemiologic evidence of rodent deaths before or at the early stage of illness and, in some cases, human-to-human transmission. 

In recent years, the most active region of the *Marmota himalayana* plague focus has been Subei County in Gansu, where animal plague is extremely epizootic and the case-fatality rate is high. However, the area is sparsely populated, only ≈15,100 residents in 66,700 km^2^, and improvements in early detection, diagnosis, and treatment have been achieved. Although the case-fatality rate is high, the disease is unlikely to spread to other areas because few persons travel outside Subei and because timely diagnosis and control help prevent further spread. Plague in the area mainly is limited to family groups, occasionally affecting neighbors or medical staff. In some years, the area had a high number of cases, but the case-fatality rate was low compared with the extremely high case-fatality rate of most years (Figure 5). Despite multiple pneumonic plague outbreaks in these epidemic spots, doctors and the public are highly vigilant and timely and effective antimicrobial drug treatment has improved patient prognoses. In 2009, for example, when an index case was identified in the county, a national medical team of experts from Beijing and Qinghai arrived at the scene to treat human plague cases and mitigate further spread (*41*).

The main transmission route of plague in the *Marmota* plague foci in China is through wounds incurred during marmot skinning, bites from infected fleas, or human-to-human transmission by pneumonic patients. In rare cases, shepherd dogs with pneumonic plague have transmitted plague to herders (*41*,*42*). In addition, Tibetan sheep (*Ovis aries*) are a host species unique to the *Marmota himalayana* plague focus and the number of human cases caused by sheep infections in this foci is second only to those caused by marmots. During 1950–2019, a total of 80 related human plague cases were reported, most caused by skinning and eating infected sheep. Tibetan sheep become infected through consuming animals that have died of plague or via bites from infected fleas. The sheep especially like chewing bone remains, which leads to oral mucosa damage and *Y. pestis* infection (*45*,*46*). Human cases have major occupational characteristics; most victims are marmot poachers, local herdsmen, and hired herders (*42*). Because of prohibitions on hunting, trafficking, and sales of marmots and related laws and education on self-protection, the number of poachers and herdsmen infection has decreased sharply. Recently, hired herders have become the most likely to be infected because they received less education on avoiding plague and engaged in skinning and eating marmots. In addition, transportation to *Marmota* plague foci has become convenient, greatly increasing the risk for long-distance transmission when persons leave foci plague infected with *Y. pestis* or carrying *Y. pestis* infected flea vectors. Illegal hunting and trading of marmots for their meat and skins also occasionally has occurred and live marmots are being shipped to densely populated cities, especially to some southern cities, where eating game animals is popular. Because of their unique geographic location and environment, *Marmota* plague foci have become excellent areas for scientific research, exploration, and tourism. Global tourists can easily reach these foci, which increases the risk for *Y. pestis* infection. Moreover, herdsmen from plague foci also can reach domestic and foreign cities within a day; thus, the increasing trend of plague transmission in densely populated areas (*47*). For example, in 2019, two patients with respiratory symptoms in the *Meriones unguiculatus* plague focus of Inner Mongolia were transferred to Chaoyang Hospital in Beijing where pneumonic plague later was diagnosed (*48*). This example demonstrates that human plague is a rare but serious infectious disease that still poses a public health risk in China and worldwide.

In summary, most human plague cases and *Y. pestis* isolates originating in the *Marmota* plague foci of China since 1950 have been concentrated in the *Marmota himalayana* plague focus. Cases from this region exhibit 3 major characteristics: the frequency of human cases during 1950–2019 slowly declined but fluctuated greatly, in sharp contrast to the rapid declines in other plague foci in China; human cases are primarily distributed in the Qinghai-Tibet Plateau and Tianshan Mountains of China; and index case-patients were mainly infected through wounds incurred while skinning infected marmots. The Qinghai-Tibet Plateau and Tianshan Mountain regions have large land areas but sparse human populations and poor or no medical facilities. Most *Y. pestis* strains derived from marmots are highly virulent (*27*), which results in outbreaks of pneumonic plague, often with higher case-fatality rates. The number of dormant foci have increased and active foci are greatly reduced in China, especially in some southern regions where one of the largest plague outbreaks occurred and primarily associated with exposure of humans to rat flea bites (*21*). In sharp contrast, the *Marmota himalayana* plague focus is sparsely populated, and the interaction between the environment, host, and *Y. pestis* is relatively balanced, which is conductive to the sustained prevalence of plague. As was the case for the 2017 outbreak in Madagascar (*8*), the outcome of human plague in the *Marmota* plague foci of China is uncertain, and risk for long-distance transmission continues, which could have worldwide public health effects. Therefore, plague prevention and control remain a strong priority in China.
